# Supplementary material for: Efferocytosis requires periphagosomal Ca2+-signaling and TRPM7-mediated electrical activity
Source: Nat Commun. 2022 Jun 9;13:3230. doi: 10.1038/s41467-022-30959-4 (PMC9184625; doi:10.1038/s41467-022-30959-4)
Supplement: Supplementary file 1 — Supplementary Information [file 41467_2022_30959_MOESM1_ESM.pdf]

SUPPLEMENTARY INFORMATION

Efferocytosis requires periphagosomal Ca^{2+}-signaling and TRPM7-mediated electrical activity

AUTHOR LIST: Michael S. Schappe[1,2], Marta E. Stremska[1], Gregory Busey[1], Taylor K. Downs[1], Philip V. Seegren[1] Suresh K. Mendu[1], Zachary Flegal[1], Catherine A. Doyle[1], Eric J. Stipes[1], and Bimal N. Desai[1,3,4]*.

AFFILIATIONS:
[1]Pharmacology Department, University of Virginia, Pinn Hall, 1340 Jefferson Park Avenue, Charlottesville, VA 22908
[2]Present Address: Department of Cell Biology, Harvard Medical School, Boston, MA
[3]Carter Immunology Center, University of Virginia, 345 Crispell Dr. MR-6, Charlottesville, VA 22908
[4]Robert M. Berne Cardiovascular Research Center, University of Virginia, 415 Lane Rd, Charlottesville, VA 22908
*Correspondence: bdesai@virginia.edu

Supplementary Figure 1

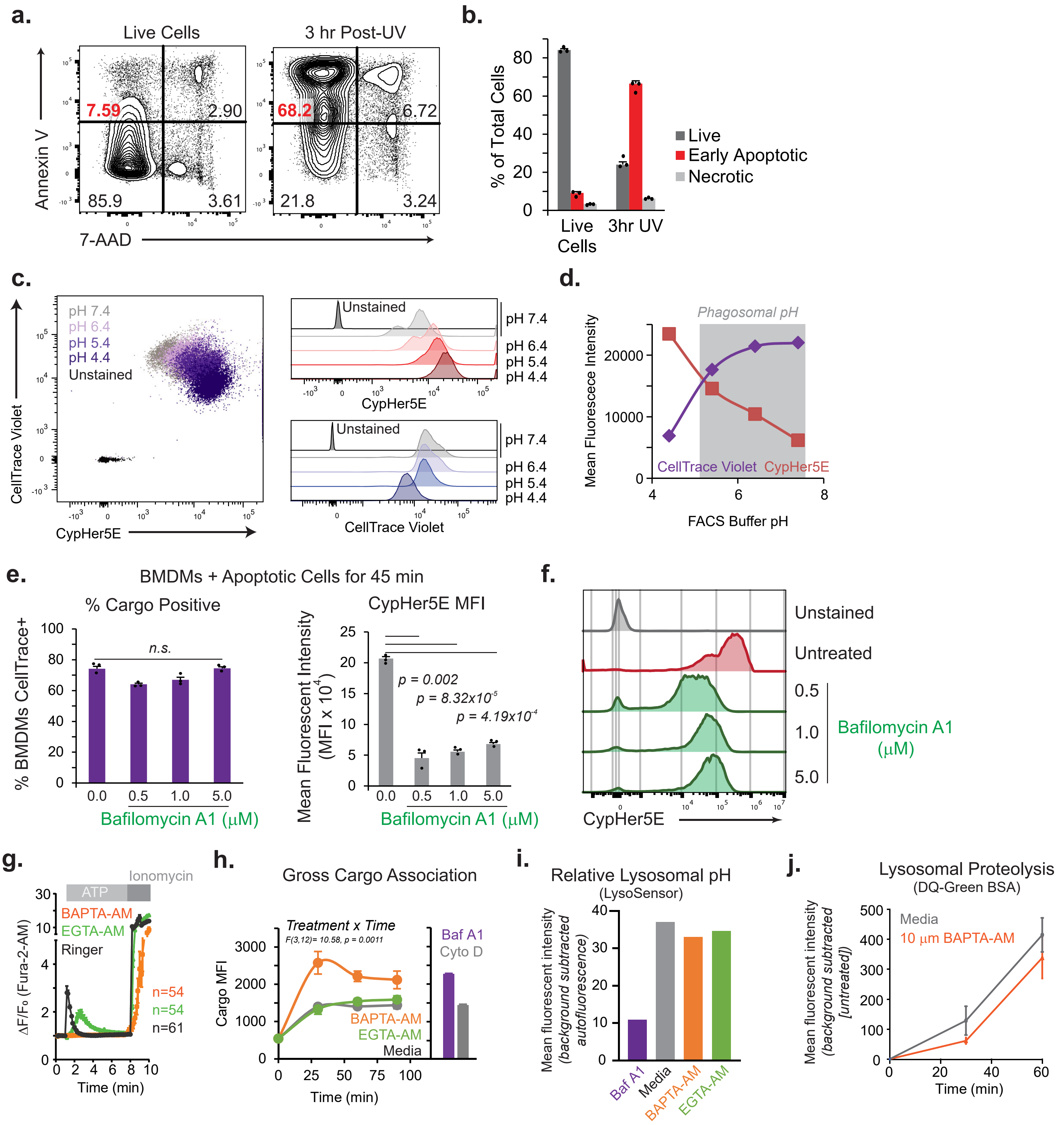

Supplementary Figure 1.

a. Live-dead staining of Jurkat cells after UV-induced apoptosis using flow cytometry. Pulse of UV-irradiation generated an increase in "early apoptotic cells" (Annexin V+, 7AAD-) 3 hr post-UV and the total cell population was used as apoptotic cell cargo in phagocytosis assays.

b. Mean percentage of cells gated as early apoptotic (Annexin V+, 7AAD-), live (Annexin V-, 7AAD-) or necrotic (Annexin V+, 7AAD+). Error bars represent SEM (n=3).

c. Flow cytometry based measurement of dye fluorescence in response to varying pH. *Left*: Overlaid cytographs of CypHer5E and CellTrace Violet-labelled apoptotic cells resuspended in buffers of indicated pH. *Right*: Histograms of CypHer5E (top) and CellTrace Violet (bottom) MFI in response to varying pH buffers.

d. Relative mean fluorescence intensities (MFI) of CellTrace Violet and CypHer5E from analysis shown in *Panel C*. The gray shaded region denotes the pH range (5.0 - 7.5) most relevant to phagosome maturation.

e. Phagocytosis of apoptotic cells in response to an inhibitor of phagosome acidification. Bafilomycin A1 was added as indicated for 15 min prior to addition of AC cargo. *Left:* Engulfing BMDMs measured by CellTrace+ cells. *Right:* Acidification in engulfing BMDMs with or without Bafilomycin A1 pre-treatment. Dots represent individual samples from individual animals (n=3) used for statistical comparison. Error bars represent SEM.

f. Representative histograms of CypHer5E MFI of engulfing BMDMs shown in *Panel e*.

g. Relative changes in $[Ca^{2+}]i$, as depicted by Fura-2-AM fluorescence, in response to ATP over time in WT BMDMs. Cells were treated with 10 μM ATP-γ-S followed by ionomycin (1 μM) as a positive control. n values shown in the figure; error bars represent SEM.

h. Flow cytometry based measurement of gross association with apoptotic cell cargo (Thymocytes) by BMDMs (T:B, 5:1) over time. Related to Fig. 1b. Engulfing CD11b+ BMDMs containing labelled cargo (CellTrace Violet) were gated and analyzed for mean fluorescence intensity (MFI) of the cargo label. Prior to collection, BMDMs were washed 3X in Ca^{2+}-free PBS and detached with 0.05% trypsin to remove unengulfed cargo from on the cell surface. Error bars represent SEM.

i. Measurement of relative lysosomal pH using LysoSensor. BMDMs were treated with indicated reagent for 30 min prior to loading with LysoSensor and measurement via flow cytometry. Values were background subtracted by the MFI of unloaded cells.

j. Lysosomal proteolytic activity measured by degradation of DQ-green BSA. Proteolytic activity was measured by flow cytometry and analyzed for MFI of DQ-green fluorescence related to cells alone. BMDMs were pre-treated for 15 min with vehicle (DMSO) or BAPTA-AM prior to addition of DQ-green BSA. Error bars reflect SD. Source data are provided as a Source Data file, and statistical testing is described in "Statistics and Reproducibility."

Supplementary Figure 2

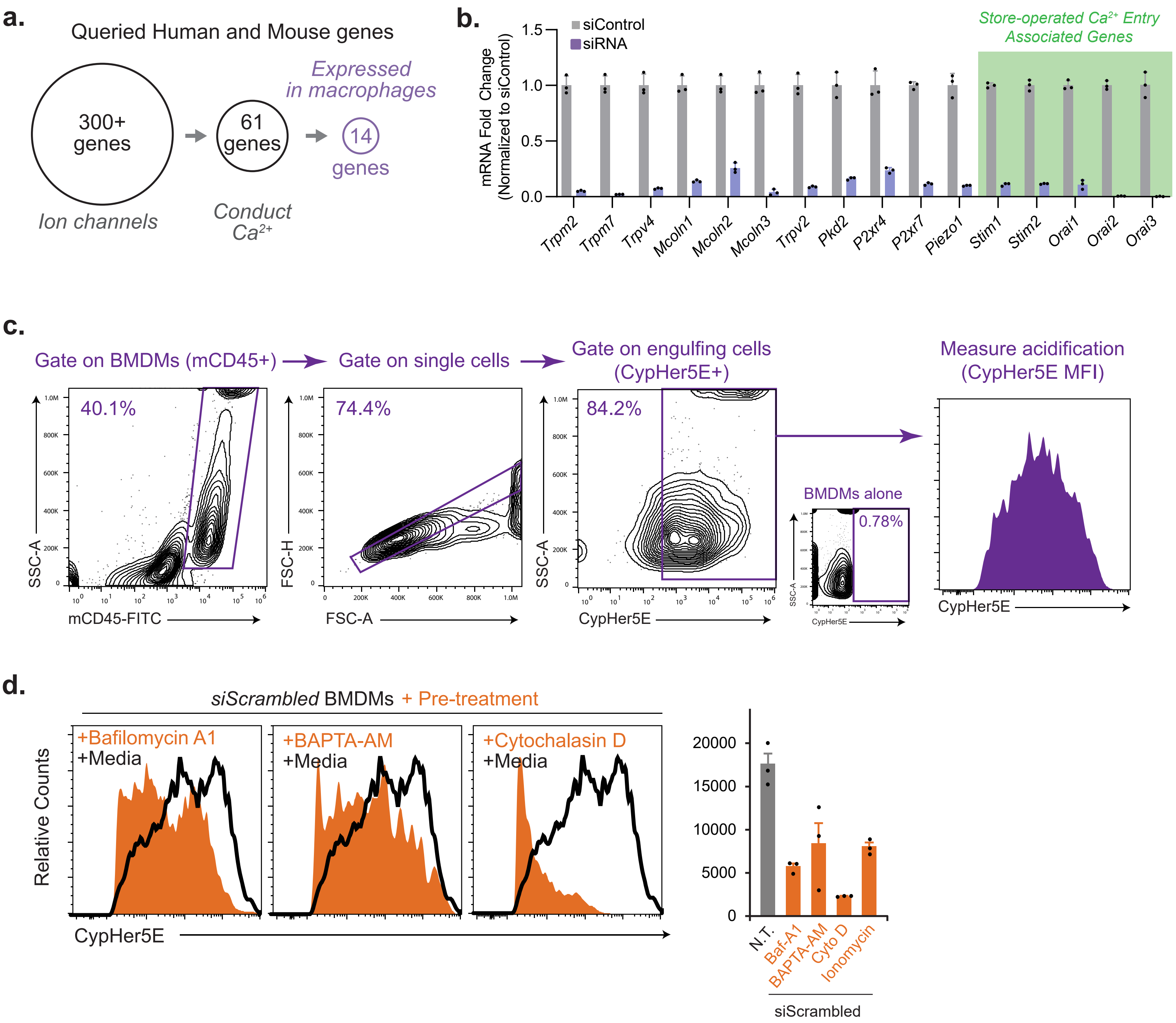

***Supplementary Figure 2*.**

a. Schematic of candidate Ca^{2+} channel genes. Relative gene expression levels used for candidate selection were determined by data available in the ImmGen RNAseq Database.

b. Gene expression analysis (qPCR) of candidate genes. Knockdown was assessed by measuring expression relative to control (siScramble-treated) BMDMs. The method is schematized in Figure 2a and detailed in Methods. Error bars represent SD centered on mean. Data represent individual samples from individual animals (n=3) used for expression analysis.

c. Flow cytometry gating strategy used in Figure 2b and 2c.

d. Representative data of control samples from siRNA channel screen. *Left:* Histograms of samples pre-treated with indicated controls (orange) compared to culture media alone. *Right:* Mean CypHer5E MFI values with error bars reflecting SD (n=3). BMDMs were pre-treated for 15 min with media alone, Bafilomycin A1 (50 nM), Cytochalasin D (100 nM), or BAPTA-AM (10 μM); ionomycin (1 μM) was added simultaneously with AC cargo. Source data are provided as a Source Data file, and statistical testing is described in "Statistics and Reproducibility."

Supplementary Figure 3

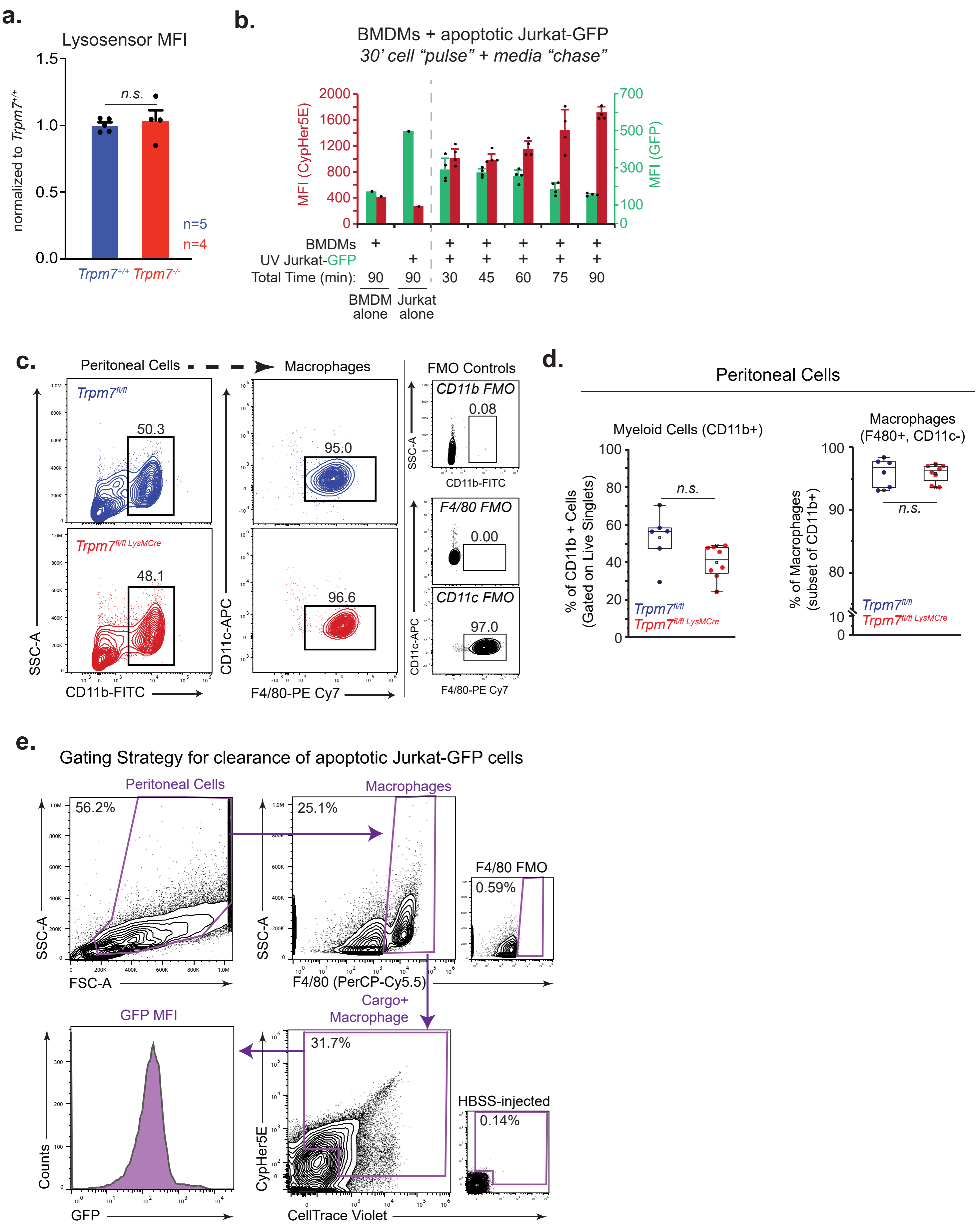

Supplementary Figure 3

a. Flow cytometry based quantification of lysosomal pH using LysoSensor, an indicator of relative lysosomal pH. Values are LysoSensor MFI normalized to *Trpm7*$^{fl/fl}$ BMDMs from indicated number of independent samples; error bars are SD centered on mean.

b. Flow cytometry based quantification of degradation of triple-labelled, apoptotic Jurkat-GFP cells by BMDMs *ex vivo*. MFI of CypHer5E (acidification, red scale and bars), and GFP (cargo protein, green scale and bars) is shown for each condition. Bar charts represent mean of n=3 independent samples; error bars are SD.

c. Representative cytographs depicting peritoneal cells and macrophage populations from *Trpm7*$^{fl/fl}$ and *Trpm7*$^{fl/fl}$ *LysM Cre* mice. Fluorescence minus one (FMO) controls are shown on the right. The data are representative of n=6 individual mice.

d. Quantification of CD11b+ and macrophage populations depicted in *Panel c*.

e. Flow cytometry gating strategy used in Fig. 4a-d. Source data are provided as a Source Data file, and statistical testing is described in “Statistics and Reproducibility.”

Supplementary Figure 4

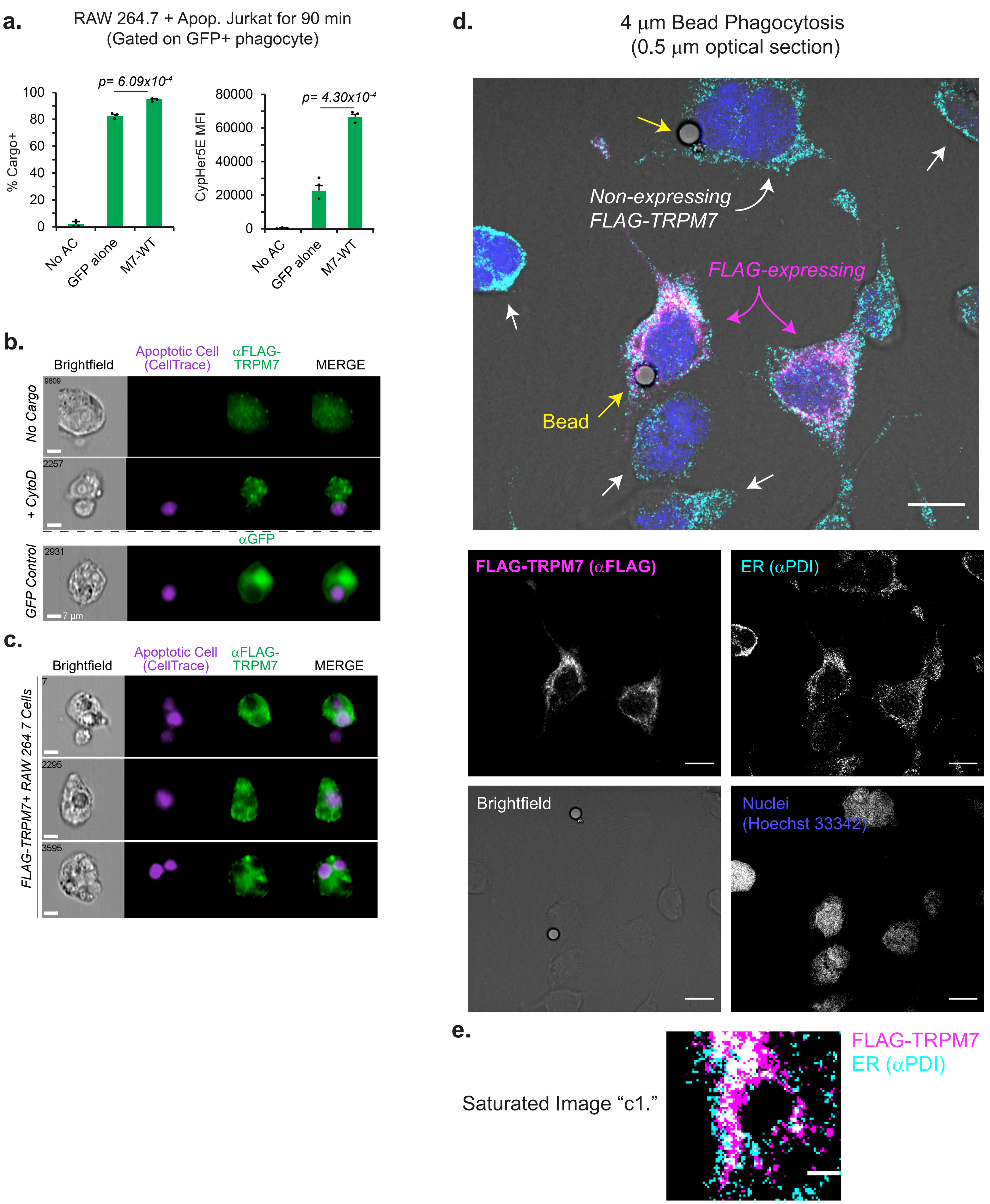

***Supplementary Figure 4*.**

a. Measurement of phagocytosis in RAW 264.7 cells ectopically expressing GFP alone or FLAG-TRPM7 +GFP. Phagocytes were transfected with GFP alone or TRPM7 and GFP for 24 hr prior to addition of apoptotic Jurkat cells (J:B = 10:1). Phagocytosis was measured in GFP+ phagocytes by flow cytometry. *Left panel* shows cargo association with GFP+ RAW 264.7 cells (CellTrace Violet+ CypHer5E+). *Right panel* shows acidification of engulfed cargo (Cypher5E MFI). Data points are mean of n=3 independent samples; error bars are SEM. Results are representative of 3 independent experiments.

b, c. Representative cells shown for treatment conditions Figure 6b.

d. *Top:* Single optical section of representative cell shown in Figure 6c with pseudo-colored merged channels. Whole field of view is shown with FLAG-TRPM7 expressing cells annotated with. *Bottom:* single-channel images are shown.

e. Merged pseudocolor shown at saturating brightness and contrast in Fig. 6c, panel c1. Source data are provided as a Source Data file, and statistical testing is described in “Statistics and Reproducibility.”

Supplementary Figure 5

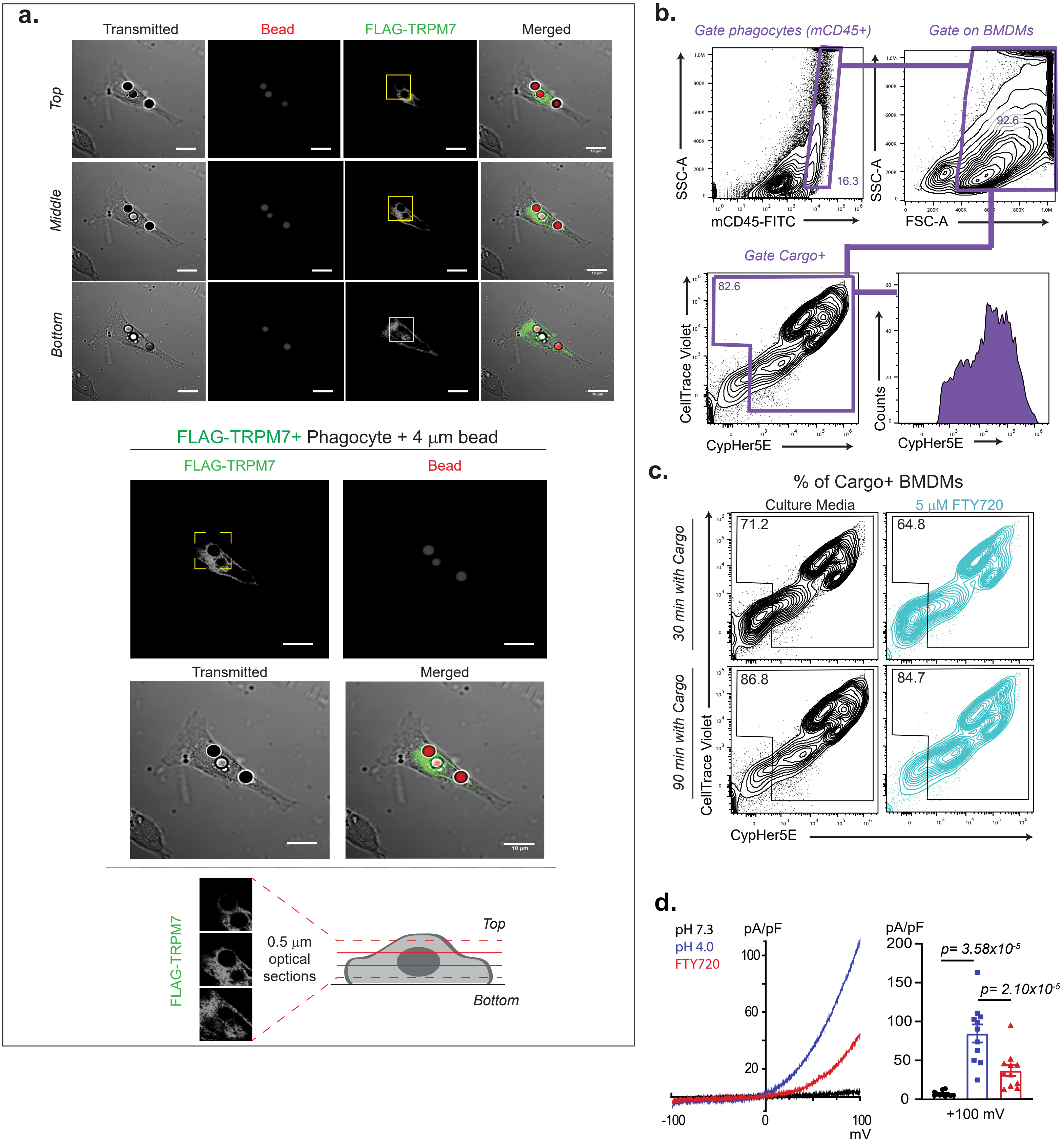

Supplementary Figure 5.

a. *Top:* Top-to-bottom z-plane optical sections of confocal immunofluorescence microscopy of FLAG-TRPM7-expressing LR73 phagocytes after addition of 4 μm beads for phagocytosis. Fixed cells were immunostained for FLAG (TRPM7-green) and fluorescent beads (red). *Middle:* Single-channel images of TRPM7, beads, and transmitted light are shown with merged pseudocolored image. Yellow dotted box indicates ROI around phagosome and is shown below. *Bottom:* ROI of same phagosome shown at three different z-planes; top-to-bottom plane is schematized on the right. Single optical sections (0.5 μm) are shown. Scale bar = 10 μm.

b. Flow cytometry gating strategy shown in Figure 7a.

c. Representative cytographs of cargo-association (CellTrace Violet +) and acidification (CypHer5E) quantified in Fig 7a.

d. Perforated-patch electrophysiology of I_{TRPM7} in RAW 264.7 cells in response to extracellular pH. *Left:* Representative I-V relationship of I_{TRPM7} as revealed in the perforated patch recording. *Right:* Quantification of current densities at +100 mV (outward current). Dots represent individual cell recordings with quantification under each condition shown. Error bars are SEM. Source data are provided as a Source Data file, and statistical testing is described in “Statistics and Reproducibility.”

Supplementary Figure 6

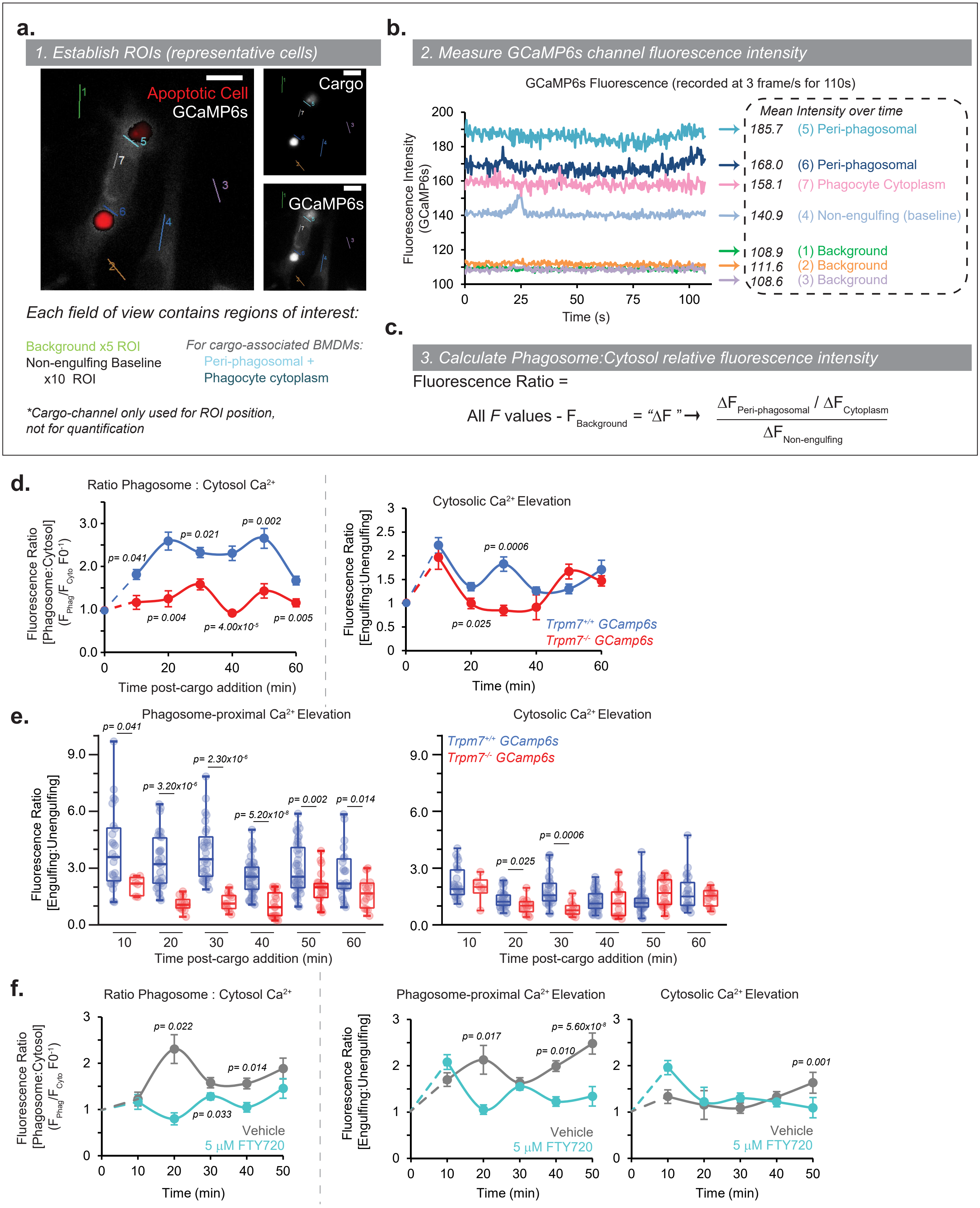

Supplementary Figure 6

a. Data analysis for Figure 9 is schematized. Fluorescence was measured via wide-field microscopy and representative cells and ROIs are illustrated for analysis shown in *Panels b* and *c*. Live cells were imaged at 37°C every 300 ms for ~2 min. Merged image of GCaMP6s (grey) and apoptotic cell cargo (red) is shown. Each field of view used for analysis contained 5 background and 10 baseline (non-engulfing cells) ROIs; each phagosome included a phagosome-proximal and cytosolic ROI. Scale bar is 10 μm.

b. Representative fluorescence intensities measured from the linear ROIs shown in Panel A. Mean fluorescence values for the ROI across the measured time course are shown on the right.

c. Formula for the calculation of fluorescence intensity ratio of Phagosomal:Cytosolic Ca^{2+} during phagocytosis.

d. Mean fluorescence ratio of changes in phagosome:cytosolic and cytosolic [Ca^{2+}] over time in WT and KO BMDMs. Data is same data set depicted in Figure 9a-b, but without the overlay of individual data points. Error bars reflect SEM (n > 20 cells).

e. Relative changes in phagosome-proximal and cytosolic [Ca^{2+}] over time in WT and KO BMDMs. Data is same data set depicted in Figure 9a and Supplementary Fig 5d (right), but with the overlay of individual data points. Error bars reflect SEM (n > 20 cells).

f. Mean fluorescence ratio of changes in phagosome:cytosolic, phagosome-proximal, and cytosolic [Ca^{2+}] over time in vehicle (EtOH) and FTY720-treated BMDMs. Data is same data set depicted in Figure 9c-d, but without the overlay of individual data points. Error bars reflect SEM. Source data are provided as a Source Data file, and statistical testing is described in "Statistics and Reproducibility."

Supplementary Table 1

List of relevant reagents and catalog information from the manuscript

Reagent	Company	Catalog #
Chemical Compounds		
BAPTA-AM	Thermo Fisher	B6769
EGTA-AM	Thermo Fisher	E1219
FTY720	Cayman Chemical	402615-91-2
ATP-y-S	Tocris	4080
Ionomycin	Cayman Chemical	56092-82-1
Bafilomycin A1	Cayman Chemical	88899-55-2
Cytochalasin D	Tocris	1233
Nystatin	Fisher BioReagents	BP2949-5
Phagocytosis Assay Reagents		
4μm FluoSpheres - Red fluorescent	Thermo Fisher	F8858
4μm polystyrene carboxylate-modified beads	Bangs Laboratories	PC05004
DQ-Green BSA	Thermo Fisher	D12050
CypHer5E	GE Healthcare	PA15405
CellTrace Violet	Thermo Fisher	C34557
Other reagents		
RPMI1640	Thermo Fisher	11875-093
Fetal Bovine Serum	RMBio	FBS-BBT
DMEM	Thermo Fisher	11965-092
HBSS	Thermo Fisher	14175-095
0.05% trypsin-EDTA	Thermo Fisher	25300-054
Paraformaldyhyde	Alfa Aesar	43368
DMSO	Sigma	67-68-5
LysoTracker Red	Thermo Fisher	L7528
LysoSensor	Thermo Fisher	L7535
Phalloidin (CF488A)	Biotium	42
TAMRA-SE	Thermo Fisher	C1171
TransIT-X2 transfection reagent	Mirius	MIR 6003
Lipofectamine 3000	Thermo Fisher	L3000015

Supplementary Table 2

qPCR Primers used for channel expression analysis

qPCR Primers<br>**Target (mouse)**	**F/R**	**Sequence (5' to 3')**
Mcoln1	F	CCA GTG TCA TGC GTT TCT GC
	R	CGA GCG GAA CTT CAC ATG GT
Mcoln2	F	CAC AGC CGC TTT TCC GC
	R	AGA CGC CAT CGC AGA TCC TT
Mcoln3	F	TCC ACA GAC TTC TGA CGG TG
	R	TCC ACT GTG AGC CTT GTT GT
Orai1	F	GAT GAG CCT CAA CGA GCA CT
	R	CCA TCG CTA CCA TGG CGA A
Orai2	F	ACA GTC AGG CCT GGT CC
	R	TGG TGG TTA GAC GTG ACG AG
Orai3	F	GCT ACC TGG ACC TTA TGG GG
	R	TGG CCA CCA TGG CGA AG
Piezo1	F	TAA GAA TAT GCT GTC GCT CCT GTC
	R	AGG CTG AAG AGC TGG ATG ACC
Pkd2	F	TCT GGA TGT TGT GAT TGT CGT G
	R	TGA AGA GCT TAA TCC AGA CCA A
P2xr7	F	GTC CCC GGC TAC AAC TTC AG
	R	TTC CTC CAG TGC CGA AAA CC
P2xr4	F	CAG CAG TGG AAT TGG GAC TGG
	R	ACC AAG AGG GTG AAG TTT TCT G
Stim1	F	AGG AGA TTG TGT CGC CCT TG
	R	GGG TCA AAT CCC TCT GAG ATC C
Stim2	F	CAC CTG CAC AGA GAA GAT AAG C
	R	ACT GCT ATC CTG GGG AGT GTT
Trpm2	F	GGA TGA CCC AAG GAA CAC AGA
	R	CAT GAG TGT GCA GGT TCT CTT
Trpm7	F	AGC AGT ATT CCA ATG ATT TTG GC
	R	TCA TAG CCA TCG TTT CAT CCT GT
Trpv2	F	GTT TGA CCG TGA CCG ACT CT
	R	GAG CCT TCT GTG TAT GCC GA
Trpv4	F	TCC TGA GGC CGA GAA GTA CA
	R	ACC ACT CTC ATC TCC AGG GG

Supplementary Table 3

siRNA Sequences

Cat. Identifier	*Gene (mouse)*	Target Sequences (siGENOME SMARTpool siRNA (Dharmacon; GE Healthcare))			
		**1**	**2**	**3**	**4**
94178	*Mcoln1*	GAACACCAUUGCCUUCCGA	UGAGAUCCCUGAUUGUUAC	CUGAUCACAUUUGACAAUA	CCUGAUACUACCUGAGAUA
68279	*Mcoln2*	GAGAGAUACCGGACUGUUA	GGACGUCGACGCUCUUUGU	GACCGUACCACGAGAAGUU	AGAAUACGAUUACCUUCGA
171166	*Mcoln3*	CCAAGAGUCUCACAAGCUA	GACUUUACGCUGACUAUAA	ACGAAACAAUUAAGCACUA	GGUGGUACAUUAUGAUCAU
109305	*Orai1*	GCACCUGUUUGCCCUCAUG	GGCGCAAGCUCUACUUAAG	CACCAAGCCUCCCGCUGAA	ACAUCGAGGCUGUGAGCAA
269717	*Orai2*	GGGCAUGGAUUACCGAGAC	UGGAACUCGUCACGUCUAA	GCGCCACAACCGUGAGAUC	GUGGAAGCGGUGAGCAACA
269999	*Orai3*	GUGACUGUCUCCCUUAGUU	GCGGCUACCUGGACCUUAU	UGGAGAACGAUCAUGAAUA	CUGUGGGACUAGUGUUUAU
18438	*P2rx4*	GUCCAGAGAUUCCUGAUAA	AUAAGUAUGUGGAAGACUA	UCAAGUCGUGCAUUUAUAA	GCUCAUCCGCAGCCGUAAA
18439	*P2rx7*	GGAAAGAGCCUGUUAUCAG	UAGCAGAGGUGACGGAGAA	UACAUUAGCUUUGCUUUGG	GGAUCCAGAGCACGAAUUA
234839	*Piezo1*	CCACCAACCUUAUCAGUGA	GCGCAAGACUGUCCUGGGA	GAAAUACAACCAUCUAAAC	CAACGGCGCAUCUUUCUCA
18764	*Pkd2*	GGAAUUGUCUGGAUGUUGU	UACGGGAGCUGGUCACUUA	CAAGAUUGAUGCCGUGAUU	CGUACAGUGGAGCGGGUUA
20866	*Stim1*	GCACCGAACUGUGGAAGUA	CGAAACAUCCAUAAGCUGA	GUGAUGAGUUCCUAAGGGA	GUGCAGUACUACAACAUCA
116873	*Stim2*	GCACAUAACUGUUGAGGAU	CAUCAGAAGUUCACAAUUG	GAUGAAAGACUUAGAGAGU	GGACGGAUGCGAUCUGGUG
28240	*Trpm2*	GAACAGCGCUUAGCCUAUG	GGACUAAGCUGGAAAAGUU	GGAGUGGGAUCCAAAGAAA	GCACUGGCCUGUAGCAAGA
58800	*Trpm7*	GCAGAAAGCUGUAGUAGAA	GAUAAUGGCUCUUGUAUUG	GGACAGAGUUUCAUUUGUA	GAAGAAACCUAGUGCUGUA
63873	*Trpv4*	GGAGAAAGGUCGUGGAGAA	GGACUGCUCUCCUUCUUGU	GACGUGAGGUGACAGAUGA	UCAAAGACUUGUUCACGAA
22368	*Trpv2*	GGCUGAACCUGCUUUAUUA	GGGCAGGCAUCACUGGUUA	UGACUGGACUGCUAGAGUA	GAAGUAAACUGGGCUGCAU
