# Supplementary material for: Efferocytosis requires periphagosomal Ca2+-signaling and TRPM7-mediated electrical activity
Source: Nat Commun. 2022 Jun 9;13:3230. doi: 10.1038/s41467-022-30959-4 (PMC9184625; doi:10.1038/s41467-022-30959-4)
Supplement: Supplementary file 3 — Description of Additional Supplementary Files [file 41467_2022_30959_MOESM3_ESM.pdf]

File name: Supplementary Data 1

Description: Original confocal z-stack of cells depicted in Figure 6c and Supplementary Fig 4d-e with adjustments for brightness and contrast. FLAG-TRPM7 is shown pseudocolored magenta; anti-PDI, cyan; nuclei, blue; and brightfield, grey.

File name: Supplementary Movie 1

Description: Phagocytosis of CypHer5E-stained apoptotic Jurkat cell by GCaMP6sexpressing BMDM. Top: GCaMP6s (Fire LUT); Middle: CypHer5E (Grey LUT); Bottom: Brightfield. Time is min:sec. Scale bar = 10 µm.

File name: Supplementary Movie 2

Description: Animated confocal z-stack of cells depicted in Figure 6c and Supplementary Fig 4d-e shown as individual channel images and single optical sections.
