# Supplementary figures and images for: Efferocytosis requires periphagosomal Ca2+-signaling and TRPM7-mediated electrical activity
Source: Nat Commun. 2022 Jun 9;13:3230. doi: 10.1038/s41467-022-30959-4 (PMC9184625; doi:10.1038/s41467-022-30959-4)

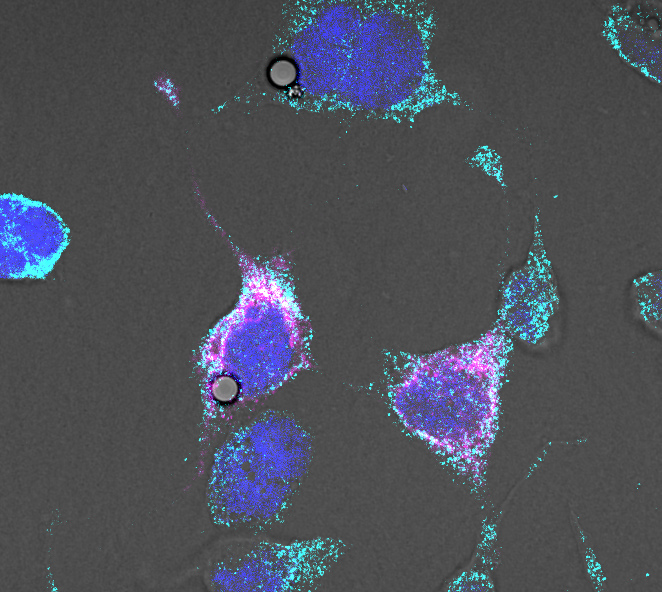

Supplement: Supplementary file 4 — Supplementary Data 1 [file 41467_2022_30959_MOESM4_ESM.tif]

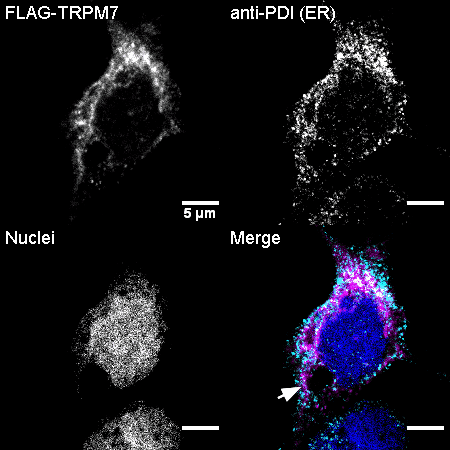

Supplement: Supplementary file 6 — Supplementary Movie 2 [file 41467_2022_30959_MOESM6_ESM.gif]
