# Supplementary material for: Efferocytosis requires periphagosomal Ca2+-signaling and TRPM7-mediated electrical activity
Source: Nat Commun. 2022 Jun 9;13:3230. doi: 10.1038/s41467-022-30959-4 (PMC9184625; doi:10.1038/s41467-022-30959-4)
Supplement: Supplementary file 7 — Reporting Summary [file 41467_2022_30959_MOESM7_ESM.pdf]

nature portfolio

Corresponding author(s): Bimal Desai

Last updated by author(s): May 2, 2022

Reporting Summary

Nature Portfolio wishes to improve the reproducibility of the work that we publish. This form provides structure for consistency and transparency in reporting. For further information on Nature Portfolio policies, see our Editorial Policies and the Editorial Policy Checklist.

Statistics

For all statistical analyses, confirm that the following items are present in the figure legend, table legend, main text, or Methods section.

n/a	Confirmed	
☐	☒	The exact sample size (*n*) for each experimental group/condition, given as a discrete number and unit of measurement
☐	☒	A statement on whether measurements were taken from distinct samples or whether the same sample was measured repeatedly
☐	☒	The statistical test(s) used AND whether they are one- or two-sided<br>*Only common tests should be described solely by name; describe more complex techniques in the Methods section.*
☒	☐	A description of all covariates tested
☐	☒	A description of any assumptions or corrections, such as tests of normality and adjustment for multiple comparisons
☐	☒	A full description of the statistical parameters including central tendency (e.g. means) or other basic estimates (e.g. regression coefficient) AND variation (e.g. standard deviation) or associated estimates of uncertainty (e.g. confidence intervals)
☐	☒	For null hypothesis testing, the test statistic (e.g. *F*, *t*, *r*) with confidence intervals, effect sizes, degrees of freedom and *P* value noted<br>*Give P values as exact values whenever suitable.*
☒	☐	For Bayesian analysis, information on the choice of priors and Markov chain Monte Carlo settings
☒	☐	For hierarchical and complex designs, identification of the appropriate level for tests and full reporting of outcomes
☒	☐	Estimates of effect sizes (e.g. Cohen's *d*, Pearson's *r*), indicating how they were calculated

Our web collection on statistics for biologists contains articles on many of the points above.

Software and code

Policy information about availability of computer code

Data collection	Microscopy - Leica Application Suite (Leica), Zeiss Zen 2.6 (blue edition; Zeiss), SlideBookPro (3i), Fluoview (Olympus, v4.1a)<br>Electrophysiology - pClamp (v9)<br>Flow cytometry - Attune NxT Software (v2.0+)
Data analysis	Flow cytometry analysis - FlowJo software (FlowJo LLC, BD; v10)<br>Imaging analysis - ImageJ (National Institutes of Health, v1.52p) and SlideBookReader (v6)<br>Electrophysiology - OriginPro (Origin; v9)<br>Statistical and Graphical Analysis - Prism (GraphPad; v8), OriginPro (Origin; v9), and Excel (Microsoft 2010)

For manuscripts utilizing custom algorithms or software that are central to the research but not yet described in published literature, software must be made available to editors and reviewers. We strongly encourage code deposition in a community repository (e.g. GitHub). See the Nature Portfolio guidelines for submitting code & software for further information.

Data

Policy information about availability of data

All manuscripts must include a data availability statement. This statement should provide the following information, where applicable:

- Accession codes, unique identifiers, or web links for publicly available datasets
- A description of any restrictions on data availability
- For clinical datasets or third party data, please ensure that the statement adheres to our policy

Source data are provided with this paper. Cuastom code was not used in this study. The IMMGEN data used for the siRNA screen are freely accessible at https://

www.immgen.org/. The methods, reagents, targets, and verified hits from this study are contained in the manuscript. In addition to the source data file, data in this manuscript and part of ongoing studies are also available upon request to the corresponding author.

Field-specific reporting

Please select the one below that is the best fit for your research. If you are not sure, read the appropriate sections before making your selection.

☒ Life sciences ☐ Behavioural & social sciences ☐ Ecological, evolutionary & environmental sciences

For a reference copy of the document with all sections, see nature.com/documents/nr-reporting-summary-flat.pdf

Life sciences study design

All studies must disclose on these points even when the disclosure is negative.

Sample size	Sample sizes are indicated in the figure legends and/or listed within the figure panel. Statistical analyses are described in detail in "Methods." No power analysis was used for sample sizes and replicates, but these were determined based on similar studies (ref. 20, 25, 31, 37, 38, 39, 42, 47, 55, 61).
Data exclusions	Data were tested for outliers using Grubbs' Test in GraphPad Prism, and outliers (<1% of measured samples, where identified) were excluded from statistical analysis.
Replication	The description of replicates is included in figure legends, where applicable. In general, all experiments were performed at least 3 times and data are represented as a composite of all experiments. In instances where representative data is shown, individual data points and/or means of the independent samples are shown, as described in the figure legends.
Randomization	Age matched mice were allocated randomly into experimental groups. All other samples were allocated randomly into experimental groups.
Blinding	Blinding was not used in the this study as the individual performing analysis was also involved in collecting and labeling samples. However, all within-experiment sample groups were run at the same time so prior knowledge had no impact on data output.

Reporting for specific materials, systems and methods

We require information from authors about some types of materials, experimental systems and methods used in many studies. Here, indicate whether each material, system or method listed is relevant to your study. If you are not sure if a list item applies to your research, read the appropriate section before selecting a response.

Materials & experimental systems

n/a	Involved in the study	
☐	☒	Antibodies
☐	☒	Eukaryotic cell lines
☒	☐	Palaeontology and archaeology
☐	☒	Animals and other organisms
☒	☐	Human research participants
☒	☐	Clinical data
☒	☐	Dual use research of concern

Methods

n/a	Involved in the study	
☒	☐	ChIP-seq
☐	☒	Flow cytometry
☒	☐	MRI-based neuroimaging

Antibodies

Antibodies used	Anti-F4/80 PE-Cy7 (Clone: BM8) Thermo Fisher 25-4801-82<br>Anti-CD11b FITC (Clone: M1/70) BD Pharmingen 557396<br>Anti-CD11c APC (Clone: N418) Biolegend 117310<br>Anti-CD45 FITC (Clone: 30-F11) Thermo Fisher 11-0451-82<br>TruStain FcX anti-mouse CD16/32 antibody Biolegend 101320<br>OneComp eBeads Thermo Fisher 01-1111-42<br>AnnexinV-7AAD Viability Staining Kit BD Pharmingen 559763<br>CD11b-Alexa fluor 488 Thermo Fisher 53-0112-80<br>Anti-DYKDDDDK Epitope Tag (FLAG) Thermo Fisher MA1-91878 and Sigma #F1804<br>Alexa fluor 488 Donkey Anti-mouse AffiniPure IgG Jackson ImmunoResearch Laboratories 715-545-150
Validation	All antibodies were validated by the manufacturers, and validation data is provided with each reagent or accessible at the product website. In general, the manufacturers test each antibody by immunofluorescent staining with flow cytometric, microscopy, or western blot analysis.

Eukaryotic cell lines

Policy information about cell lines

Cell line source(s)	Jurkat (Clone E6-1 (ATCC® TIB-152™)) and RAW 264.7 (ATCC® TIB-71™) from American Type Culture Collection (ATCC)<br>LR73 and Jurkat-GFP cells from the Lab of Kodi Ravichandran (UVA; currently, WashU)
Authentication	Microscopic inspection and expression of surface markers by flow cytometry
Mycoplasma contamination	Tested cell lines were negative for mycoplasma contamination
Commonly misidentified lines<br>(See ICLAC register)	No commonly misidentified lines were used in this study.

Animals and other organisms

Policy information about studies involving animals; ARRIVE guidelines recommended for reporting animal research

Laboratory animals	Mice used followed procedures approved by the Animal Care and Use Committee of the University of Virginia and adhering to National Institutes of Health Animal Care and Use Guidelines and. Mice were housed in 12 hr light-dark cycle at room temperature (20 to 23°C) with 40 to 70% humidity with ad libitum access to food and water. Male and female mice aged 7 to 14 weeks were used for all experiments. Trpm7fl/fl and Trpm7fl/fl<br>(LysM cre) mice were generated as described previously. Trpm7fl/fl (CX3CR1 Cre) mice were generated through crossing Trpm7fl/fl mice to B6J.B6N(Cg)-Cx3cr1tm1.1(cre)Jung/J (Jackson Laboratories; 025524) to subsequently generate Trpm7fl/fl and Trpm7fl/fl (CX3CR1 Cre) mice on a mixed background. GCaMP6s-expressing strains were generated crossing established mouse strains with the B6;129S-Gt(ROSA)26Sortm96.1(CAG-GCaMP6s)Hze/J mice, which contain a LoxP-flanked STOP codon.
Wild animals	Study did not involved wild animals
Field-collected samples	Study did not involve samples collected from the field
Ethics oversight	Animal experiments were approved by the Institutional Animal Care and Use Committee (IACUC) at the University of Virginia and adhering to National Institutes of Health Animal Care and Use Guidelines

Note that full information on the approval of the study protocol must also be provided in the manuscript.

Flow Cytometry

Plots

Confirm that:

☒ The axis labels state the marker and fluorochrome used (e.g. CD4-FITC).

☒ The axis scales are clearly visible. Include numbers along axes only for bottom left plot of group (a 'group' is an analysis of identical markers).

☒ All plots are contour plots with outliers or pseudocolor plots.

☒ A numerical value for number of cells or percentage (with statistics) is provided.

Methodology

Sample preparation	Macrophages were plated on 24-well non-coated tissue culture plates at a density of 0.1 x 10^6 cells/well overnight prior to phagocytosis assays. At the end of the experiment, cells were washed 3X with cold PBS and incubated with 0.05% trypsin-EDTA for 5 min to remove unengulfed cargo and detach macrophages. Cold culture media was added to inhibit trypsin activity and cell suspensions were then collected (1500 rpm for 5 min), washed 1X with PBS, and resuspended in FACS Buffer. With Jurkat cargo, mCD45-FITC (Clone: 30-F11; 0.5 ug/ml) was used to further discriminate macrophages from cargo; with thymocytes, CD11b-Alexa fluor 488 (Thermo Fisher; Cat# 53-0112-80; 0.5 ug/ml). Instrument voltages and gating was established based on unstained macrophages, unstained cargo, and single-stained fluorescent controls (with 0.01% HCl added for CypHer5E positive control). Gating strategy is described in Supplemental Figures. For in vivo measurements of phagocytosis, cells were recovered via periotneal lavage in saline, recovered cells were pelleted, resuspended in ACK lysis buffer for 5 min (red blood cell lysis), washed 1X in cold PBS, and stained for flow cytometry. For peritoneal phenotyping, cells were stained with TruStain FcX anti-mouse CD16/32 (5 microgram/ml; Biolegend; #101320) for 10 min at 4°C prior to addition of the fluorophore-conjugated antibody cocktail for 30 min. As shown, antibodies used for staining were anti-mouse CD11b FITC (Clone: M1/70), F4/80 PE Cy7 (Clone: BM8), and CD11c APC (Clone: N418) at 0.5 microgram/ml. Cells were then washed 2X in FACS Buffer (0.5% BSA, 2 mM EGTA) prior to analysis. All experiments included single stain controls using OneComp eBeads (Thermo Fisher; #01-1111-42) and fluorescence minus one controls with cells for gating
Instrument	Attune NxT (ThermoFisher)

Software	Collection: Attune NxT Software (ThermoFisher)<br>Analysis: FlowJo software (FlowJo, LLC BD)
Cell population abundance	No sorting was used for this study.
Gating strategy	All cells were first gated on FSC/SSC according their spectral properties. Unstained samples and fluorescence minus one (FMO) samples were used to set up negative control gates. Identity of the cells was confirmed by staining with appropriate surface markers.

☒ Tick this box to confirm that a figure exemplifying the gating strategy is provided in the Supplementary Information.
